# Thermophiles reveal the clues to longevity: precise protein synthesis

**DOI:** 10.20517/jca.2021.38

**Published:** 2022-01-18

**Authors:** Manisha Deogharia, Priyatansh Gurha

**Affiliations:** Center for Cardiovascular Genetics, Institute of Molecular Medicine and Department of Medicine, University of Texas Health Sciences Center at Houston, Houston, TX 77030, USA

## Abstract

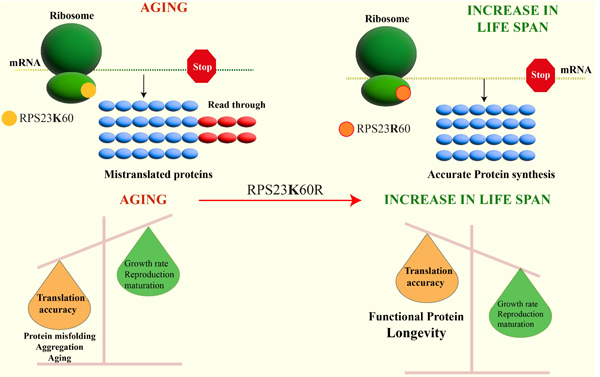

All living organisms are classified into three domains - Bacteria, Archaea and Eukarya. Archaea are generally classified as extremophiles even though they are adapted to nearly all environments. In many instances, Archaea more closely resemble Eukarya, and some of their proteins often have no apparent homolog in bacteria. Studies in archaea have contributed to the identification of many evolutionary conserved fundamental mechanistic discoveries related to histones and ribosomes, RNA modification, DNA replication, transcription, and gene organization to name a few. Therefore, it is not surprising that the mystery of aging is now revealed in archaea. The hyperthermophilic archaea reveal that the clues of longevity lie in precise and error-free protein synthesis^[1]^.

Aging is a natural process that results in the progressive physiological and functional loss of tissues and organs, thereby increasing the organism’s risk of mortality. Factors that contribute to the aging process, include genomic instability, epigenetic changes, mitochondrial dysfunction, stem cell exhaustion, and the loss of proteostasis^[2]^. Recent studies have shown that the capacity of many cells and organs to maintain proteostasis under diverse conditions declines with age, and unsurprisingly, proteostasis loss is implicated in the etiology of a wide variety of human diseases linked to aging^[3,4]^. During the lifetime of an organism, proteins are constantly exposed to stressors that impair their function. Protein homeostasis networks have evolved to monitor and regulate the synthesis, folding, trafficking, and degradation of proteins. The earliest process in the protein life cycle that modulates cellular proteostasis is translation. Changes in levels or mutations in the components of translational machinery have a significant impact on longevity in several animals. For instance, mutation or depletion of ribosomal proteins and translation factors extend lifespan in yeast worms and flies^[5,6]^. Similarly, blocking the mTOR (mammalian Target of Rapamycin) system through rapamycin reduces protein synthesis and increases lifespan.

While the significance of protein synthesis in the aging process is widely accepted, it is unclear whether errors in protein synthesis or translational fidelity may play a part in aging per se. Protein synthesis error rates in bacterial systems are estimated to be as high as 1 in 10^3^ per amino acid^[7–9]^. According to this estimation, up to 15%−20% of the cellular protein pool may contain mistranslation errors. Mistranslated proteins may not have a significant impact on cellular proteostasis at a young age, due in part to the quick turnover and efficient clearance of cellular proteins. However, in the older animal, protein turnover rates, proteasome activity, and autophagy decline, making them more sensitive to error-prone protein translation. Therefore, mistakes in protein translation have the potential to cause a variety of age-related diseases^[10,11]^. So far, however, it was unclear whether accurate translation can affect cellular aging and if the rate and extent of mistranslation increase with aging.

In a publication by Martinez-Miguel *et al*.^[1]^ the authors propose a mechanism of aging that is highly dependent on translational accuracy. The authors study ribosomal protein RPS23, a homolog of prokaryotic S12 protein that is conserved in all three domains of life, and implicate it in the maintenance of protein translational accuracy. The ribosomal protein S12 (eukaryotic homolog RPS23) contributes to translation accuracy by assisting with tRNA selection, codon-anticodon pair evaluation, and rearrangements of the 30S subunit required for aminoacyl-tRNA accommodation^[12,13]^. S12 mutations that confer resistance to streptomycin (induces protein translation error), show reduced levels of miscoding. Distinct changes in the S12 protein affect translation accuracy with some that promote miscoding while others reduce decoding errors^[13]^. Thus, changes in this critical protein could affect decoding accuracy.

Martinez-Miguel *et al*.^[1]^ demonstrate that a single amino acid alteration in a ribosomal protein present in archaea can improve metazoan translation fidelity and survival. The authors conducted a comprehensive phylogenetic analysis of RPS23 and discovered that a lysine residue at position 60 is evolutionarily conserved, except in hyperthermophilic archaea, where arginine replaces it (RPS23-K60R). *CRISPR* gene editing was used to insert a mutation into the Drosophila melanogaster *Rps23* gene, which led to the substitution of the lysine 60 with an arginine residue. The translational accuracy of mutant and wild-type flies was determined with a reporter construct that contained a renilla luciferase gene followed by a firefly luciferase gene separated by an in-frame linker sequence containing a stop codon. Inclusion of a stop codon produces firefly only if a read-through had occurred, and thus served as a measure of translational accuracy. It was discovered that the K60R mutation in the RPS23 protein decreased the frequency of mistakes made during protein synthesis, thereby leading to a reduction in firefly luciferase production in the mutants. Specifically, the stop-codon read-through increased considerably with aging in the control flies, but not in the RPS23-K60R mutant.

The authors also examined if the K60R mutation had the same effect in other species and whether it affected the total rate of protein production as well. Similar results were obtained in *Caenorhabditis elegans* and *Schizosaccharomyces pombe* (yeast), where the expression of the RPS23-K60R protein also reduced stop-codon read-through. Surprisingly, increased fidelity was not related to alteration in global protein synthesis rates in flies and worms as measured by the incorporation of puromycin in proteins and expression of translation marker protein peIF2a or pS6K and p4E-BP. Puromycin is a tyrosyl-tRNA analog that inhibits translation by tagging and releasing polypeptide chains from translating ribosomes.

Given that the variant (RPS23 K60R) was initially discovered in hyperthermophilic archaea, the authors contended that the protein should provide an evolutionarily conserved mechanism of heat tolerance. Indeed, flies, worms, and yeast expressing RPS23-K60R protein were able to grow and survive at temperatures above the optimum. Finally, the physiological benefit of this mutation was demonstrated by an increase in healthy lifespan. The authors demonstrate that when the mutation was introduced, it improved lifespan by 9%−23% in all three model organisms. Additionally, the authors extend their findings by noting that treatments that prolong lifespan (such as rapamycin, torin, and trametinib) do so by enhancing translational fidelity in controls but not in mutant K60R strains. Thus, they concluded that the primary determinant of the aging process was a reduction in translational fidelity.

Protein synthesis fidelity is inextricably linked to energy expenditure, such that cells will optimize accuracy; both too little and too much accuracy will have a detrimental effect on organismal growth and reproduction. Accordingly, in this study, the RPS-K60R mutant flies and worms developed more slowly when compared to their wild-type counterparts. This was not connected with decreased fecundity or behavioral changes in general. However, the precise mechanism by which developmental delay or heat tolerance occurs remains unknown.

As is the case with every discovery, the study generates a plethora of new queries. The most pressing question is whether discoveries in worms - which have a lifespan of 2–3 weeks and around 2 months for flies - can be extended to humans.

Heterozygous missense mutations in the *RPS23* gene (R67K and F120I) are associated with brachycephalic, trichomegaly, and developmental delay. When we searched the Genome Aggregation Database (gnomAD) for 76,156 genomes, we could identify no change in RPS23 (matching to K60). Furthermore, the K60R mutation was anticipated to be pathogenic (Polyphen2: http://genetics.bwh.harvard.edu/pph2). As a result, research that precisely describes the role of this mutation and the situations under which it is tolerated will be required to extend the findings to humans.

Similarly, the mechanism of action of RPS23 K60R is currently unclear. While the authors propose that it is essential for translational accuracy, they note that the mutation may alter the structure of the ribosomal RNA, resulting in a conformational transition of the 30/40S ribosome, which may lead to translational accuracy. It is worth noting that both lysine and arginine are positively charged basic amino acids that take part in electrostatic interactions^[14]^. However, the guanidinium group in arginine permits interactions at more than one orientation, thereby allowing for a greater number of electrostatic connections than lysine^[14]^. Exact biochemical and structural information is now awaited to test this hypothesis.

We envisage that since lysine(s) in proteins are targets of a bevy of post-translational (PTM) modifications which could be abolished or gained with an arginine substitution and can be a possible mechanism as well contributing to the function of RPS23. Our bioinformatic analysis found that acetylation was the most likely PTM at this lysine in RPS23 protein.

These findings also open a new avenue of investigation for cardiovascular researchers into the relevance of translation fidelity in disease pathogenesis and cardiovascular aging. For example, chronic pathogenic stress results in ventricular hypertrophy, necessitating *de novo* protein synthesis to augment cardiomyocyte size. It will be intriguing to explore whether a similar mechanism that improves translational accuracy influences the course of cardiac hypertrophy and heart failure.

Misfolded protein oligomers, unfolded protein response activation, and abnormal protein aggregation have been observed in the hearts of patients suffering from hypertrophic cardiomyopathy, nonischemic cardiomyopathy, or dilated cardiomyopathy^[15]^. As a result, it will be interesting to see if inducible activation of RPS23-K60R in adult hearts can improve translational fidelity related mechanisms that can help alleviate disease pathology in mouse models of these diseases.
